# COVID‐19 and the Unequalizing Infrastructures of Financial Inclusion in Tamil Nadu

**DOI:** 10.1111/dech.12674

**Published:** 2021-08-16

**Authors:** Isabelle Guérin, Vincent Guermond, Nithya Joseph, Nithya Natarajan, Govindan Venkatasubramanian

## Abstract

This article discusses the impact of the COVID‐19 pandemic on microfinance borrowers in Tamil Nadu, India. Through an examination of the social and financial infrastructures underpinning inclusive finance, the article demonstrates how the COVID‐19 pandemic exposes the limits and exclusionary tendencies of the for‐profit financial inclusion industry. The unequalizing breakdown of financial inclusion infrastructures during the pandemic prioritizes future revenue extraction over current livelihood needs, throwing hard‐hit borrowers back on hierarchical informal financial and social infrastructures to cope with COVID‐19‐induced risk. Tracing the experiences of poor microfinance borrowers in Tamil Nadu, this article examines how COVID‐19 is reshaping inclusive financial infrastructures in ways that reveal the dynamics of exclusion at the heart of financial inclusion.

## INTRODUCTION

Veena Mary, a Dalit Christian widow, has been highly stressed about her five microfinance loans and numerous ceremonial obligations (*moi*) in recent months.^1^ ‘The anxiety about my debt has made me sick’, she said. ‘My heartrate has risen but I have not gone to the hospital yet. If I go to the nuns here, I will have to spend 100 rupees on treatment’.^2^ On 24 March 2020, Prime Minister Narendra Modi announced a national lockdown with four hours’ notice for the country. Like tens of millions of internal migrant workers, Veena Mary's sons returned home as their jobs ceased, leaving her with a significant reduction in household income. Two days later, the Reserve Bank of India announced a three‐month moratorium on loan repayments, including microfinance loan and interest repayments, later extended for another two months. This was a relief, since it allowed Veena Mary and many others not to worry about these repayments, but also a source of concern, as the moratorium also blocked any further borrowing.

Many microfinance providers, however, did not respect the moratorium. From mid‐May 2020, almost three months before the end of the moratorium, loan officers returned and started pressurizing Veena Mary to repay, reminding her that not only did interest continue to accrue, even though delays had been permitted, she would also be charged interest on the interest. She would end up having to pay much more on each loan.^3^ Once the moratorium was lifted, repayments became mandatory, which left her no choice but to hide from loan officers, sitting still for hours on collection days to give the impression that no one was home.

At the same time, the central government's promised pandemic relief, in the form of cash transfers, which was supposed to be distributed efficiently through banks, largely missed its target, precisely because of the dysfunctional banking infrastructure. Although Veena Mary has a bank account, opened specifically to access social welfare programmes, she received nothing. Like other villagers, the only forms of help she was able to secure were the food distributed and cash transfers in the form of cash from the state of Tamil Nadu. Indeed, some of her neighbours even saw their meagre revenues captured by the automatic repayment of loans.

Veena Mary is served through infrastructures of financial inclusion and these have been hailed as a key means of support for India's working poor, both during the first wave of the pandemic and the subsequent national lockdown, and afterwards (Rawat, 2020). Yet, as the reality on the ground shows, during the first lockdown and in the months that followed, microfinance — through the continued presence of loan officers during the moratorium, and the digitization of payments — was primarily employed as a tool of capturing precarious asset streams, not distributing financial support.

As is often the case in times of crisis, social and relational infrastructures have been resilient where formal structures have failed. Veena Mary has used a number of informal avenues to access financial support during the lockdown. She has borrowed from neighbours, her supervisor (also Dalit but much wealthier) at the government school where she works as a cook, and pledged jewels loaned back to her by her son‐in‐law. This was primarily to repay other debt, including to microfinance institutions. The rigid system of ceremonial gifts and counter gifts, that builds on and feeds relationships of mutual support and solidarity, has also played a role in deepening her financial distress at this time. While the gifts she received for her daughter's marriage had greatly helped at the time, as we write this in January 2021, she is now obliged to return them despite the crisis. Although Veena Mary started skipping meals and using wood instead of gas in order to save money, rather than meeting her own needs, she still had to borrow to pay her ceremonial debts. She also approached several people she knew to ask for a large, lower‐interest loan to repay her microfinance and other loans. However, having exhausted her entire social network to help her meet previous needs, she has not succeeded.

Contrary then to claims of financial support and resilience, Veena Mary's case offers a glimpse into how infrastructures of financial inclusion, promoted by the Indian state (Mader, 2014) and globally (Roy, 2010), create ‘immiserizing’ dynamics. Financial inclusion as a poverty alleviation strategy is on the ascent, promising to democratize access to forms of credit for the poorest (Erturk et al., 2007). Yet in March 2020, during a moment of profound reproductive crisis, as the income of the working poor in India was abruptly curtailed by the national lockdown, the infrastructure of inclusion suffered a significant breakdown, as the moratorium prevented access to new resources for those in need. Focusing on this moment of breakdown, this article aims to ‘excavate’ the hidden politics behind financial inclusion processes which usually remains invisible (Graham, 2010: 3).

Understanding the devastating effects of the pandemic on livelihoods requires exploring the gap between the promises of financial inclusion infrastructures and the way in which they are implemented and experienced, as well as the way in which they resist or break down in the face of crisis, how they ‘include’ but also ‘exclude’ certain segments of the population, and how they are both constitutive of and shaped by social and power relations. Financial ‘inclusion’ is manifested across inclusive banking, microfinance loans, and even state relief rolled out through women's savings groups, all of which constitute the overlapping of state and commercial entities providing liquidity to the poor and undergirded by complex, everyday social relations of informal lending.

In examining inclusive financial infrastructures during a period of breakdown, we advance two arguments that problematize the portrayal of financial inclusion as a form of crisis alleviation and access to global financial resources for the poor (El‐Zoghbi, 2019). First, our investigation of the power relations behind financial inclusion suggests that the responses to economic disruption in India in the year following the first lockdown, starting end March 2020, conceal an undercurrent of exclusion, with access to liquidity running dry for the most vulnerable amid ongoing demand for repayments. In our examination of the response of the microfinance industry, and its impacts on borrowers when the economic and financial situation of the latter becomes more volatile and uncertain, we demonstrate how financial inclusion operates as a means by which capital is able to capture future income among the working poor.^4^ Job losses, financial relief measures that frequently fail to reach those most in need, and short‐lived moratoria on debt repayments together with sustained pressure of microfinance loan officers have increased rather than ameliorated income insecurity for poor women like Veena Mary.

Second, in our exploration of what happens in lieu of access to new microfinance loans, we show that infrastructures of financial inclusion are deeply entangled with and sustained by relations of informal finance, including money lenders, mutual help and ceremonial gift‐giving. In many instances, such informal lending channels allow for greater control over repayment schedules for the working poor, thus offering more flexible avenues of relief. Yet we remain ambivalent about the emancipatory potential of these so‐called ‘informal’ financial relations, as they in turn are riven with hierarchies of caste, class and gender, again resulting in a deeper crisis for the poorest. Failure to repay loans carries a high price, risking exclusion from social groups and relations of support. As such, we find that caste and class‐based inequalities have increased as a result of the pandemic. This occurs in two ways; first, those from Dalit communities who already face the greatest levels of debt, job insecurity and harassment from lenders (Mosse, 2018) see a worsening of their position. Second, new forms of inter‐caste lending create additional channels of caste‐based exploitation by upper‐ and middle‐caste groups. Whilst we do not focus explicitly on gender dynamics, the majority of Indian microfinance debtors — 99 per cent in 2019 (Sa‐Dhan, 2019: xv) — are women. Thus, our analysis is implicitly gendered, given our overwhelming focus on female respondents.

Our argument is primarily based upon qualitative research in three villages in central‐east Tamil Nadu, an area where two of the authors of this article have been working for two decades. Pre‐existing social relationships with respondents have enabled us to follow the impact of the pandemic through repeated telephone interviews and then in real‐life, face‐to‐face interactions. From June to November 2020, phone interviews were conducted monthly, with 60 households. Additional face‐to‐face interviews, including group discussions and interactions with a number of loan officers, took place until March 2021. As with all qualitative surveys, the objective was not to achieve a representative sample but to ensure a diversity of situations in terms of caste, gender, occupation, asset holding and debt sources. Overall, 140 interviews were conducted with 75 people, with between one and seven interviews per person. Given the situation — loss of income for most of the respondents, cost of recharging the phone — the respondents received a financial compensation of INR 1,000 (US$ 14). Interviews were supplemented by document analysis of key blogs, policy briefs, reports and newspaper articles relating to the current state of the microfinance industry and its response to the COVID‐19 pandemic, both globally and in India.

We proceed by engaging with the literature on inclusive infrastructure which, we argue, allows us to investigate the breakdown of financial inclusion during the COVID‐19 pandemic in India and beyond. Next, we discuss how the microfinance industry reacted to the pandemic at the international level, before moving on to our case study. We start by examining the history and complexity of financial inclusion infrastructure in Tamil Nadu, before looking at how this infrastructure proved exclusionary and enabled increased capture of revenues in times of COVID‐19. Finally, we turn to the social infrastructures that undergird these formal financial arrangements and discuss their dubiousness as a fallback social safety net.

## FINANCIAL INCLUSION: INTERROGATING INFRASTRUCTURE

Research on everyday debt relations has highlighted how broader financial systems are reproduced in varied, even contrary ways, through the ‘sticky materiality of practical encounters’ (Tsing, 2005: 1), complicating the representation of a seemingly monolithic and powerful debt infrastructure of financial inclusion. With regard to microcredit and over‐indebtedness, the ethnographic evidence highlights the multiplicity of processes that constitute the infrastructure of loan disbursement and collection, which in turn depends on the diversity of local political and moral economies, including the social and moral meaning of debt (Guérin, 2014; Kar, 2018).

The seeming infallibility of structures of inclusion is therefore punctured in ways that reveal the need for greater attention to the materialities and social relations which constitute inclusion. For example, as Green (2019) highlights, valuing land as collateral for microfinance in Cambodia is a process of construction, rooted in the social relations of the valuing officer, and of the family in question. Guermond (2020: 235) shows how the creation of remittance markets and the incorporation of remittance flows and households into microfinance circuits constitute contested and contingent projects that require ‘extensive financial, material, technological, legal and discursive construction and, importantly, behavioural engineering’. James’ (2015) focus on the social relations that underpin financial inclusion in South Africa highlights the explosion of over‐indebtedness as an ambivalent process, with many debtors also being creditors and managing to earn money ‘from nothing’. These accounts contribute to an understanding of the infrastructures of financial inclusion as always in the making, precarious and fragile — especially in times of crisis. The problematization of infrastructures as a ‘terrain and object of political contestation’ (Elyachar, 2017: 50) enables us to interrogate financial inclusion through a lens that takes politics and social relations more seriously. Such work builds on and complicates Foucault's (1998) thesis on infrastructure as a technology of governance, to elucidate the multiple politics shaping infrastructures, the ways in which they are (re)produced through social relations, and how they lead to varied and unequalizing effects. We draw out two key areas of thinking in developing our arguments in this article.

### Infrastructure, the Capture of Time and Space, and the Devolution of Risk

One of the most significant characteristics of credit/debt is ‘its ability to link the present to the past and the future’ (Peebles, 2010: 226). The ambiguity of this dyad stems from the fact that while credit may constitute a promise about the future, it also poses the risk of capturing the debtor's present and future time if the repayment of the debt becomes problematic. As demonstrated in the above vignette, debt for Veena Mary and many other Tamil villagers no longer serves as an instrument in ‘future making’ (Green et al., 2012: 1641), rather, it has become precisely what prevents her from projecting herself into the future.

The critical literature on financial inclusion, and particularly on rising debt‐led development, offers a robust account of how the expansion of so‐called ‘inclusive’ financial infrastructures has enabled finance to capture the future labouring time for precarious workers through advertising and expansion of credit and other financial products (Roy, 2010), such as increasing access to bank accounts and the digitization of (re)payments (James, 2015). Such studies also demonstrate how the risks of financial instruments are devolved onto debtors (e.g. Mader, 2014). For lenders and investors, risk management involves complex chains of financial intermediation (ibid.). For precarious populations, it is crucial to rely and build on social infrastructures to manage the risks involved in their financial inclusion (Kar, 2018).

However, the relentless requirements of finance are often at odds with the insecurity and precarity that characterize income streams of the poor. As Saiag (2020) has shown in Argentina, the time demands of finance in the form of credit repayments are not always reconcilable with the time patterns of work, particularly in instances where people are precariously employed. This resulted in a feeling of losing control over one's time due to credit taking — where finance, and not the individual, ‘dominates the time of labour’ (Saiag, 2020: 24). Put simply, the ceaseless demands of debt repayment rendered those with insecure incomes even more precarious, increasing their exposure to risk. In view of the impact of the pandemic, we therefore ask: what happens when the infrastructure's demand over time among precarious households is rendered more uncertain through the increased insecurity of work? What does this mean for the financial infrastructure, and for the reshaping of risk for those served by it?

### Beyond Physical Infrastructure: The Centrality of Social Relations

Infrastructures are far more than the physical elements which constitute them materially, rather, they are also made up of dynamic social relations which (re)shape how they function. As Berlant (2016: 393) argues, ‘[i]nfrastructure is not identical to system or structure, as we currently see them, because infrastructure is defined by the movement or patterning of social form. It is the living mediation of what organizes life: the lifeworld of structure’. In a similar vein, Anand's (2017: 6–7) analysis of water infrastructure in Mumbai takes us beyond material infrastructure, to think about how ‘water infrastructures are generative of a multiple, entangled, non‐constitutive outside to the form and performance of the liberal city’. The seeming simplicity and success of water pipes and the water flowing through them are only part of the story when understanding the working of infrastructure. In addition, Anand (ibid.: 10) discusses Mumbaiites’ everyday engagements with the politics and infrastructure of water to materially and ideologically coax water through pipes which in turn transforms them, forging a kind of ‘hydraulic citizenship’ — referring to the ‘social relations through which everyday political claims are recognized’.

To further understand these ‘lifeworlds’, we carefully examine the role of informal social infrastructures of finance in supporting ‘formal’ financial inclusion infrastructures. McFarlane and Vasudevan (2014) highlight how such informal infrastructures are often deemed undesirable and even criminalized — yet they are essential to the day‐to‐day (re)making of urban life. This is certainly evident from the arguments provided in India for expanding access to financial inclusion. Formal lending is juxtaposed with the maligned alternative: ‘usurious money lenders’ who are ‘continuing to exploit the poor’ (GoI, 2014: 3). In addition, expenditures and gift‐giving practices during ceremonies held to celebrate life‐cycle events are often portrayed in the mainstream economic and policy‐oriented literatures as informal, wasteful and ostentatious practices (e.g. Banerjee and Duflo, 2011).

Yet, as Guérin (2014) has shown in the context of South India, formal and informal lending overlap, with forms of informal lending in certain instances even seen as favourable by the working poor, as they allow more flexibility in terms of repayment schedules. Formal loans are linked in multiple ways to informal loans. They are repaid through debt relations structured along caste, class and gender lines, and shaped by patronage, employment, kinship and reciprocity. Furthermore, informal practices of ritualized exchange, such as ceremonial gifts, produce relational value and have therefore the potential of being highly ‘productive’ in the future (Elyachar, 2005). These relationships are not just ‘out there’, ready to be used as a resource; rather, they are ‘situationally produced and performed’ (ibid.: 145) and require huge amounts of time, labour and resources to be maintained. In rural Tamil Nadu, ceremonial exchanges represent the main form of accumulated and circulated wealth, while savings bank accounts remain empty (Guérin et al., 2019). This arguably more flexible and malleable relational infrastructure is much more attractive than the rigidity of formal financial infrastructure. With the crisis flowing from the pandemic, however, this relational infrastructure is being put to the test, as we shall see below. While we highlight some of the ‘generative’ capacities of such informal social infrastructures, we also shed light on their darker side (Meagher, 2010) and explore the hierarchies imbued with and constitutive of the new social relations emerging as a result of the crisis. We also show how ‘inclusion’ creates new dynamics of exclusion (Meagher, 2015).

## MICROFINANCE INFRASTRUCTURE AND THE COVID‐19 PANDEMIC

To examine the infrastructure of financial inclusion as a political process, we take as entry point the industry's response to the COVID‐19 pandemic. The pandemic and the resulting global recession have put the current microfinance model in jeopardy through simultaneously endangering the regular income flows of microfinance institutions (MFIs) and their access to capital (Ogden, 2020). Aware that the widespread losses of employment and income would prevent borrowers from repaying loans made to them, microfinance institutions dramatically curtailed their lending to mitigate the risk of loan defaults. The World Bank's Consultative Group to Assist the Poor (CGAP) Global Pulse Survey showed that two‐thirds of the surveyed MFIs had ‘slashed lending by more than half compared to normal levels’ (Zetterli, 2020). While microloans have long been portrayed as a tool of resilience by financial inclusion advocates (Bernards, 2020), vulnerable households have lost access to this apparent ‘crisis remedy’ at a point of crisis in resource access, further exacerbating the complexities of household reproduction in this period. Whilst a briefing from CGAP (2020: 2) to microfinance regulators suggests that remaining ‘pro‐poor’ should be the first guiding principle in the development of crisis measures, and ‘preserv[ing] the safety and soundness of microfinance providers’ only the fourth, the current collapse in lending and concurrent access to liquidity for the poorest suggests that lenders are adopting a more inward‐looking approach. Instigated by the microfinance industry in order to protect the sector, the drastic curtailment of lending activities at the global level contributed to undermine the resilience of poor borrowers.

Furthermore, breakdown threatens to deepen livelihood insecurity for many. Ignoring numerous calls for loan waivers, a significant portion of microloans have been rescheduled and restructured globally, including in regions where moratoria have been placed on loan disbursement and collection (Sotiriou, 2020). There are serious concerns among the microfinance community about how these restructured loans will perform once the moratoria are lifted (Meagher, 2020; Sotiriou, 2020). As a result, much of the focus to deal with the current crisis has been on how to maintain ‘credit discipline’ (Tarazi, 2020) and ‘the repayment culture among microfinance borrowers’ (Sotiriou, 2020). According to the World Bank, relief policies should only be targeting those borrowers with strong payment records, and not what they call ‘wilful defaulters’ — those who can repay but refuse to do so — and ‘zombie borrowers’ — those who struggled to repay before COVID‐19 and are unlikely to repay at the end of the moratoria (Dijkman and Salomão Garcia, 2020).

What the crisis and concurrent curtailment of lending activities have thrown into sharp relief is the almost impossible trade‐off between providing meaningful relief to borrowers and maintaining the security of the microfinance industry. As the World Bank guidance on borrower relief measures in relation to COVID‐19 signals, regulators are strongly advised to obtain a thorough understanding of the financial impact of relief measures prior to adoption (ibid.). Relief to borrowers is pitted against the sustainability of the sector, thus recasting the most precarious borrowers, arguably those who are most in need, as too high‐risk. In a CGAP COVID‐19 briefing, it was noted that ‘beneficial as borrower relief measures may be, they risk weakening repayment discipline and masking the true financial position of MFP [Microfinance Providers] portfolios’ (Meagher, 2020: 6). As a result, while forms of loan rescheduling — such as moratoria, often with interest accrual^5^ or even interest capitalization^6^ — and restructuring have constituted the policy norm (Bernards, 2020; Rhyne and Dias, 2020), expanded relief mechanisms such as, for instance, debt forgiveness seem not have been part of the discussions.

The burden of the breakdown of financial inclusion infrastructure is therefore largely borne by precarious borrowers. In many cases, the interruption of lending activities is a direct result of MFIs trying to minimize their total non‐performing assets in the long run, so as to limit decreases in profit. Ultimately, what seems to be at issue is the continuous mortgaging of borrowers’ future incomes to meet the present needs of lenders and investors, leading to a disconnect between the functioning of financial inclusion infrastructures and the need of borrowers to be included.

## DEBT INFRASTRUCTURES IN TAMIL NADU

India has a long history of pro‐poor lending in its development trajectory. State, commercial and social debt/credit infrastructures in Tamil Nadu consist of a web of complex and entangled socio‐economic relations, which are comprised of numerous actors with varied political and economic interests, power and incentives, and imbued with a combination of solidarity and social hierarchies.

With its origins in the colonial era, institutional lending to the rural poor expanded considerably between the 1960s and the late 1980s. Rural branches of national banks increased by 40.6 per cent between 1969 and 1990 (Shah et al., 2007), and a national lending programme was set up to target the rural poor. Yet by the late 1980s, largely due to a combination of political capture and structural poverty, millions of rural Indians had defaulted on their state‐backed loans, with state banks suffering significant losses leading to wide sectoral instability. This instability contributed to the demise of existing state‐backed rural credit schemes in the early 1990s, in line with the deregulation and privatization of national banking and financial services promoted by the Narasimaham Committee in 1991 (ibid.). The subsequent rise of informal credit — which had steadily been declining in the decades following independence in 1947 — prompted policy makers to implement various measures, the result of which is a complex financial infrastructure present in the most remote villages.

Key amongst the measures taken to promote pro‐poor lending was the creation of Self‐help groups (SHG), the first microcredit model in India, supported by international donors, NGOs and national banks, which enabled groups of people, mostly women, to save and borrow money among themselves. These groups allowed women to borrow at rates lower than most other informal loans, and to profit from lending to each other. The standard rate of 2 per cent per month (24 per cent per year), though high, is considered acceptable since the interest returns to group members. Importantly, well‐functioning groups could borrow from banks at even lower rates, usually under 12 per cent per year. While only state banks could initially lend to SHGs, over time, commercial banks also became interested in this new niche market. Granting these groups access to forms of credit that would normally be inaccessible, SHG constituted a social infrastructure of financial inclusion that became legible to formal finance.

SHGs enjoyed a golden age in the early 2000s, then began to decline in favour of commercial microfinance granted by for‐profit organizations (Nair, 2010). Since then, the microfinance sector's loan portfolio witnessed rapid and sustained growth, increasing in value from INR 225,440 in 2010 (US$ 5 billion at the March 2010 exchange rate) to INR 1,080,750 million (approximately US$ 15 billion) in March 2020 (Sa‐Dhan, 2010, 2020). By 2019, 87 per cent of Indian microfinance clients were indebted to for‐profit MFIs, and 84 per cent of total outstanding microfinance loans in India were owed to for‐profit MFIs (Sa‐Dhan, 2019: xv). Moreover, the focus on financial services (savings, credit) was also expanded toward the broader concept of ‘financial inclusion’, which notably includes the distribution of government cash transfers through bank accounts. In 2017, 80 per cent of Indians had a bank account, an increase from 53 per cent in 2014 (Ravi, 2019). While direct deposits of cash transfers through bank accounts are supposed to improve transparency and eliminate malpractices, these programmes, as we argue below, continue to prioritize political interests and rivalries between national and state programmes that are a source of permanent confusion and, above all, dysfunction. Other measures allegedly put in place to protect debtors and facilitate transactions and transparency, including the creation of a credit bureau and the automatic deduction of repayments, also tend to work mostly in favour of creditors. Crucially, this multi‐layered and complex infrastructure of financial inclusion — constituted by state banks, commercial banks, MFIs and different types of SHGs — is also deeply enmeshed with and supported by a heterogeneous range of informal credit relations. It is thus necessary to look at the micro scale to grasp the specificity of local financial landscapes and the way they shape, and are shaped by, unequal social and power relations.

As elsewhere in rural India, villages in the Cuddalore, Villupuram and Kallakurichi districts of Tamil Nadu, where this research is located, remain deeply segmented according to caste. The *Ur* (settlement) is the term used for the main village, where rights to reside are reserved for non‐Dalits. It is internally segmented by caste, here comprising mainly Vanniyars, who can be considered middle caste, and Naidus and Reddiyars, who are part of the upper castes. Located at some distance from each village is a separate hamlet to which residences of the Dalit castes are restricted, which is referred to as the ‘colony’. However, the region has undergone profound changes over the last decades, due to the combined effects of economic growth, decline of agriculture, migration and debt. Class distinctions are expressed not only through the differentiated ownership of assets (land, tractors, livestock, etc.) but also the uneven access to regular or permanent non‐agricultural jobs. The poorest have to settle for daily agricultural (*coolie*) work and increasingly, for men, non‐agricultural work outside the village. For the landless, the main employment opportunities constitute a permanent trade‐off between regular but seasonal jobs (e.g. sugar cane harvesting and brick moulding) based on a wage advance system and involving dire working conditions, and casual jobs (e.g. manual labour in construction, transport and markets). However, for some young people, who are increasingly qualified, including Dalits, slightly better jobs in manufacturing and services are now accessible. These class distinctions overlap partly but not completely with caste. While some, although not many, casual workers are among the upper and middle castes, a number of Dalits have experienced relative upward mobility, often as labour intermediaries. Among the Vanniyar middle castes, class positions are very mixed. And, as elsewhere in India, female employment has been declining steadily over the last few decades in all social categories. The available occupations remain very limited: land cultivation for those whose family owns land, and agricultural day labour and MGNREGA (a state‐run public works programme) for the others.

While employment has diversified, it has remained volatile and precarious. At the same time, needs have constantly increased, due to both the commodification of social reproduction (e.g. health and education) and growing aspirations (e.g. housing, ceremonies). Crucially, debt has been, and continues to be, a response to this discrepancy. Constituting a historical component of rural livelihoods, debt has dramatically expanded in recent decades in Tamil Nadu, albeit with unequal conditions of access (Guérin et al., 2020).

Almost all households in the surveyed villages now have a bank account, a precondition to access cash transfers. However, only asset owners and high wage earners are eligible for bank loans, which remain the cheapest credit option (between 8 and 12 per cent interest per annum). Some non‐farm wage earners without a formal contract now have access to these bank loans, with automatic deduction from their bank account for repayment acting as a guarantee. While SHGs represent another channel for bank loans, especially for women, the size of loans tends to be much smaller and a regular savings capacity — unequal along class lines — is required. In our three villages, 29 state‐run SHGs are active and 25 are non‐Dalit, located in the *Ur*, illustrating the strong bias of access to this particular financial inclusion infrastructure in favour of the upper castes. Finally, because they do not require collateral and prior savings, and are often cheaper than most (not all) informal loans, microfinance loans, with a 2 per cent average monthly rate, are very attractive, even if the rigidity of repayment schedules does not sit well with income volatility. Most households, through women, now use them (around 80 per cent in our sample), and often combine several providers (up to seven, with an average of 2.6 per household).

Incorporation in microfinance networks is highly differentiated along gender, class and caste belonging. Before the pandemic, women from the upper and middle castes/classes were more likely to be members of well‐functioning SHGs and not to borrow from MFIs, while Dalit women were more likely to borrow from for‐profit MFIs and not to be SHG members. In addition to these ‘formal’ financial options, men and women juggle many other sources of credit, including loans from pawnshops and local elites (most often from middle and higher castes), labour recruiters (most often from low and middle castes) as well as family, friends and neighbours (Guérin, 2014). Oscillating between exploitation and solidarity, the terms of these informal options vary widely with regard to cost, repayment terms and penalties for default. Regardless of these conditions, they remain unavoidable not only to meet needs and compensate for the rigidity of formal loans but also to maintain and cultivate social ties. In other words, informal credit options fill the gaps in the formal financial inclusion infrastructure, allowing precarious populations to build viable relations with these formal systems. As we demonstrate below, ceremonial debts play a central role in the intermingling of finance and social relations.

## FINANCIAL INFRASTRUCTURES IN TIMES OF PANDEMIC: SELECTION, EXCLUSION AND CAPTURE

By tightening their borrowing selection criteria and focusing on the (sometimes automated) capture of very meagre incomes through collection of loan repayments in response to the pandemic, both the state and for‐profit MFIs have reinforced the exclusionary nature of financial inclusion infrastructures. While protecting financial interests, such processes significantly aggravated stress for borrowers and compelled precarious women to literally hide from loan officers. Meanwhile, public relief infrastructures, offering emergency loans and digital social benefits, also proved to be selective and exclusive. Political competition between federal and state programmes and strict assessments of creditworthiness contributed to further exclude the most vulnerable.

### Covid‐relief Measures and Cash Transfers

On 26 March 2020, two days after the announcement of the national lockdown, the central government issued a COVID‐19 relief package, many components of which relied on financial inclusion infrastructures. A cash transfer of INR 500 (US$ 7) per month was to be made for three months to all women's bank accounts created through the Pradhan Mantri Jan‐Dhan Yojana (PMJDY) financial inclusion scheme (translating as the Prime Minister's Scheme for People's Financial Welfare) initiated under Narendra Modi's premiership in 2014. Senior citizens, people with disabilities, and widows were promised two instalments of INR 1,000 to these same accounts. Amongst our respondents, no one had received, or knew anyone who had received, these cash transfers in their bank accounts. A key reason was that their bank accounts had been opened not through this specific PMJDY scheme, but prior to 2014, to receive payments from the NREGA employment programme. Participants did not know where to go to claim this transfer either, as it came from the central government with no local body as intermediary.

At the national level, it is estimated that 38 per cent of poor households and 46 per cent of rural households have been excluded from the transfer programme as they did not have a female member holding a PMJDY account (Somanchi, 2020). These accounts are a key part of the flagship PMJDY financial inclusion scheme. This shows the lack of coordination between federal and state programmes that is typical of Indian social policies, reflecting centre‐aligned political interests which are a major cause of exclusion in parts of the country. In addition to the PMJDY scheme provided by the central government, the Tamil Nadu state government offered a one‐off cash payment of INR 1,000 through the Public Distribution System which provides food grains in the village on a monthly basis. In addition to food assistance for five months, all study respondents received this sum making this distributive infrastructure more reliable.

During the pandemic, already‐existing digital transfers also became more difficult to access. Several participants did not receive their pensions, NREGA wages and farmer subsidies. While they said that this was a regular occurrence, they were not even permitted entry to the administrative offices to make a complaint. Even for those who received their transfers, money still had to be converted into cash. Yet both ATMs and bank branches are difficult to access. This is especially true for women who very rarely have access to motorized two‐wheelers or bicycles. In two of our surveyed villages, they had to travel 5 and 16 km by foot while avoiding police checks. Going to a bank branch did not even guarantee a withdrawal, as was also observed in other parts of India (Khera, 2020), since banks only permitted a few dozen customers to enter each day.

While distribution of social benefits through the banking system proved rather ineffective, the same cannot be said about the digital ‘capture’ of debt repayments. Prior to the crisis, several educated young men amongst our respondents, including Dalits, were receiving wages directly in their bank accounts and had therefore been eligible for bank loans, with repayments being automatically deducted. While many of them lost their jobs during the lockdown, the automated deduction continued, dramatically curtailing their access to liquidity. Direct deduction from bank accounts also took place when people were issued fines. This extension of digital infrastructures of extraction during the pandemic contributed to worsening the precarious situations of many households.

### Credit through Self‐help Groups

As part of the relief package, the finance minister also announced the doubling in value of collateral free loans to SHGs — from INR 1 million to 2 million (from US$ 13,800 to 27,600) per SHG, claiming this would benefit 70 million households through 6 million SHGs. In our study region, the potential of any measure targeting SHGs remains very limited due to the disappearance of most NGO‐driven SHGs on the one hand and, on the other hand, the uneven spread of any existing state‐run groups between and within villages. Crucially, only one SHG qualified for loans of the value of the previous credit limit (INR 1 million), with the average value of bank loans to SHGs in India amounting to INR 0.25 million (US$ 3,450). At the national level, the proposed change would only benefit a very few groups which already qualified for much higher credit.

In the village with the well‐functioning state SHGs however, the Panchayat^7^ was allocated INR 400,000 (US$ 5,500) by the state government. This sum was to be shared between all 23 groups with over 300 members, yet loans ended up being issued to 12 women only, nine of whom were from the upper and middle castes. The three Dalit women chosen to receive the loans, amongst the 30 women of their caste who were SHG members, were those with assets. Other Dalit women were told that they were not eligible because they did *coolie* (wage) work and did not have a *tholiyal* (enterprise), thus reinforcing the unequal provision of this very limited COVID‐19 relief measure along class and caste lines.

### Microfinance and Moratoria

As mentioned above, the Reserve Bank of India (RBI) allowed a three‐month moratorium on principal and interest payments for all pre‐existing term loans, which was then extended for five months, until the end of August 2020. MFIs were mandated to allow repayments to be delayed, without any impact on ratings by the Credit Information Bureau (RBI, 2020). This offered significant relief, albeit only temporarily.

Before the pandemic, women reported running helter‐skelter on the day fixed for the monthly repayment of their MFI loan on a regular basis, as they struggled to borrow the sum they needed to pay the instalment due. They were used to loan officers sitting outside their house for hours. Before the pandemic, a common saying among women we spoke to was that loan officers would insist on repayment even in case of a death in the family: ‘They collect their money over the corpse, saying “first you pay this and then you cry”’.^8^ When MFI activities in the villages completely ceased, women were relieved as they no longer had to face this constant pressure to repay. However, the disappearance of MFIs from the village also meant that they stopped issuing new loans, including emergency loans. This was partly in order to avert a liquidity crisis and was consistent with national and international accounts of the microfinance sector's response to the crisis. Between March and June 2020, lending activity in India decreased by 96 per cent compared to the same quarter of the previous year. Activity then picked up again; between June and September 2020, lending activity represented 43 per cent of the previous year's activity (*Times of India*, 2020).

Availing of the moratorium had serious long‐term financial implications. While lockdown‐affected borrowers needed relief from financial pressures, microfinance providers were in dire need of liquidity to remain solvent and maintain legitimacy to investors. MFIs were permitted to allow interest to accrue during this period. As mentioned above, the collection of compound interest on outstanding microfinance payments was initially permitted. As a result, when loan officers returned to the villages in June 2020, they could legitimately threaten that borrowers would be charged ‘interest on the interest’ if they did not repay. Systematic attempts at coercing borrowers to make repayments despite the moratorium, under threat of escalating interest charges, highlight the tendency of MFIs to privilege their needs and those of investors over the needs of borrowers.

In addition to coercive practices used by loan officers, some women reported facing pressure from other microfinance group members who were able to make payments during the moratorium. Solvent co‐borrowers were concerned that they would have to bear the cost of delays and possible defaults of those unable to make payments. The joint liability mechanism used by MFIs to devolve the work of extracting payments to women themselves thus became an additional extractive mechanism in times of crisis.

Some borrowers organized collectively to assert their right not to repay during the moratorium. Others decided to repay to avoid interest accrual and capitalization. Based on interviews with borrowers and loan officers as well as on group discussions, a clear distinction between Dalit and non‐Dalit borrowers emerged. Many non‐Dalits decided to pay back, often by borrowing from other sources. As for Dalits, most were unable to secure additional credit without having to agree to excessive interest rates. Consequently, they decided to wait and hope that MFI interest would be lower than the alternatives.^9^

When microfinance providers started to lend again, borrowing conditions had tightened, even for customers who had previously proved their creditworthiness. Coming back to the case of Veena Mary, one of her microfinance providers now required a bi‐weekly repayment for any new loans, which she could not afford. According to her, this technique was used to better manage risk and discourage borrowers whom they anticipated were likely to face difficulties due to rising income insecurity. At the same time, even those eligible for new loans were wary of borrowing from MFIs when income in the immediate future remained uncertain. The unequalizing breakdown of microfinance stretched well beyond the moratorium as the economy remained stalled. Anticipating, and perhaps already responding to, aggressive debt collection practices by some of its members, the Microfinance Institutions Network reiterated the importance of ‘fair interactions with borrowers’ as part of its post‐moratorium guidelines (*Economic Times*, 2020). Yet, when the moratorium ended many loan officers demanded repayment and intensified their use of pre‐pandemic coercive practices to enforce the non‐negotiability of delays. Most women either pawned their own gold ornaments or borrowed elsewhere to repay. Those who were able to repay without pledging assets were mainly non‐Dalits as well as a few wealthy Dalits. One‐third of borrowers we spoke to were forced to default on at least some monthly payments after the moratorium. They borrowed to make payments some months and missed others saying they simply could not pay. For instance, Parvati, a Dalit woman from the colony in Pudur who had missed one instalment, said*, ‘*I told them to charge as much interest on this as they wanted, and I will pay it later. I have no money at all now’.^10^

Those who defaulted reported persistent harassment from loan officers, with instances of officers going to women's worksites and shouting at them in the presence of their employers. One woman said that a loan officer threatened to call everyone in the village and humiliate her in front of them. Another woman reported having received a call from a loan officer telling her that her house would be seized if she did not repay. The strategy that proved most effective in securing returns, however, was the linking of loans to the credit bureau. For example, one loan officer told one of our participants: ‘Fine, don't pay us, but remember you won't be able to get a loan from a single other source’.^11^ In India, all formal lenders — including MFIs but also banks and gold loan companies — use biometric details to check credit histories and will not issue new loans to people with a listed overdue payment. Women are very much aware of this. Vanitha from the small middle caste hamlet in Pudur said: ‘If we want to organize a protest in our village we only need five minutes [i.e. this can be easily done], but if we don't repay our loans then we'll never be eligible for any in the future’.^12^

Over time, MFIs found new ways of ensuring loan recovery by introducing a refinancing option. They started issuing small top‐up loans, most of which would be used to clear past outstanding payments to be then repaid in full on a new schedule. For borrowers, this meant that their negative credit ratings could be reversed, allowing them to access credit from other sources. This further pledging of future incomes constituted one of their only options to ‘buy time’.

## SOCIAL AND RELATIONAL INFRASTRUCTURE AS GENERATIVE?

The unequalizing breakdown of financial inclusion infrastructure during COVID‐19 revealed the relative resilience of social infrastructures underlying them. While almost all informal borrowing and lending, including ceremonial debt, also came to a standstill immediately at the start of the lockdown, these informal circuits re‐activated much more quickly. We contend that such informal social relations can certainly be ‘generative’, and so too can the debts which arise from them. Yet the crisis also conjured up the inequity of debt relations and the asymmetry of positions between creditors and debtors. Among the most vulnerable were not only those with few or no assets or streams of income, but also those with limited or exhausted social networks, or who were on the wrong side of informal financial cycles, especially those who had just married off their daughters. We argue that the generative nature of the social infrastructure therefore remains ambivalent and in constant need of (re)negotiation.

### Informal Lending

In the early days of the lockdown the breakdown of reciprocal exchanges for low or no interest was most pronounced for the marginalized. As peer solidarity drastically tightened within ever smaller circles, hierarchical solidarity was often the only option. We heard repeatedly that ‘corona closes the doors’, not only to preserve social distancing but also because circuits of mutual aid and credit — the cement of local sociability and economy — were seriously weakened. While an extension of networks could be observed during demonetization, the COVID‐19 crisis produced the opposite reaction.^13^ For those who relied on a daily wage to meet their subsistence needs, the lack of employment meant that they sometimes simply had nothing to lend. As the brick kiln migrant worker, Elangovan, told us: ‘It is not even possible to see money with our eyes’.^14^ Anxiety about the future also constricted relationships; lending to those with no assets and whose ability to sell their future labour had become very uncertain proved too risky. Most villagers recounted a climate of permanent suspicion, especially at the beginning of the lockdown. Even good friends avoided each other because of the potential embarrassment of being solicited and having to refuse. Those seen as rich ‘quarantined themselves’ to avoid the virus and unwanted solicitations.

This narrowing down of relationships was expressed in numerous ways. Some women confessed to immediately eating what they had cooked because they felt it was harder to refuse to share a meal than it was to refuse unprepared food. Some people stopped going out altogether. Although mutual help between distant relatives and friends broke down, it is worth noting that reciprocal exchanges via strong ties (among close kin, friends or even co‐workers) continued. This was for instance the case for Arun, a young Dalit man, whose sister pawned her gold so that he could look after his baby. Among young men and women sharing a workplace, interest‐free loans were also frequent. Sarada, for instance, lent to her co‐worker at school, borrowing from her mother who lives in Bangalore. Conversely, the intensity of need and desperation also led to attempts to capture and steal from loved ones. This was the case of Sudha who admitted having taken her niece's anklets during a visit in order to pawn them.

As cash became scarce, the cost of collateral‐free borrowing from local moneylenders, most often from middle‐ and high‐caste local elite, sometimes from wealthy Dalits, increased drastically in all the studied villages. While monthly interest rates for informal sources rarely exceeded 5 per cent prior to the pandemic, they reached 10 or even 15 per cent during the crisis. For many households without assets, survival was only possible through these very expensive informal credit channels, which continued to be available when microfinance was absent. More than half of the households we met had to pledge assets or sell them directly. Gold, which normally plays the role of a quasi‐currency, was often what people pledged first, before title deeds, vehicles, or even insurance savings account. Families who had nothing to pledge sold trees, livestock as well as household equipment.

The scale of the crisis led some to accept unprecedented arrangements. For instance, Baladasan, a young Dalit, had to pledge his motorbike for a few days to a upper‐caste trader for a credit of INR 1,000 (around US$ 14) in order to buy food. Virtually strangled by funeral expenses which occurred during the lockdown, he successively had to pawn his motorized vehicle, then sold part of his livestock to recover his vehicle, which he eventually lost. Importantly, while a number of villagers were losing their assets, others were more secure. For example, one Naidu (high caste) woman continued to receive her salary (INR 4,000 or US$ 55 monthly) as a kindergarten assistant in a government school. She also knew that, if needed, she could count on her brothers who are engineers in Bangalore. When asked in June if she had pledged her gold, she asked: ‘Who would pledge gold when there are no agricultural expenses?’.^15^ She seemed unaware that others were forced to pawn their gold for food.

### Ceremonial Exchanges

Beyond informal debts, the resumption of ceremonies, combined with the insistence on compliance with a structured and rigid ceremonial gifting system, had contrasting effects. While it allowed some to access liquidity on favourable terms during the pandemic, it also reinforced the precariousness of others. Alongside their social and symbolic significance, ceremonies are financially significant. Ceremonial gifts are meticulously accounted for; hosts of ceremonies keep notebooks in which they list gifts received whereas guests check their own books before they attend celebrations in order to determine the value of the gift they need to give. Gifts made to others constitute savings while gifts that are received represent debts that have to be repaid in the future. Depending on their position in the ceremonial cycle and their number of daughters (the dowry represents an important part of ceremonial expenses), households are either creditors or debtors within these chains of reciprocity (Guérin et al., 2019). As Elyachar (2005) argues, such social systems constitute key relational infrastructures which are reliant upon extensive and continued maintenance. Significant time is spent on organizing and attending events, consolidating the sums to be gifted by the required date (including through borrowing), and determining the appropriateness of gifts one receives relative to what was owed by the giver.

Microfinance loans are deeply intertwined with ceremonial expenses. More than half of our survey respondents reported having taken the loans that they were repaying during the pandemic to finance their own ceremonies or to contribute to those of close relatives. During the early stages of lockdown, most ceremonies were cancelled, resulting in significant budget relief for many. When ceremonies resumed, people were left with complex calculations and trade‐offs to make. Postponing a life‐cycle ceremony meant delaying the recovery of capital disseminated in the form of gifts, carrying the risk that the accumulated gifts in the past may never be returned. At the same time, organizing a ceremony soon after the lockdown could result in receiving relatively little compared to what had been gifted in the past. Those who decided to organize ceremonies did not hesitate to knock on doors to claim their due. This social infrastructure offered them a form of financial security that enabled them to control the temporal aspects of (lack of) credit access by calling back their savings. Conversely, for those with large sums to repay, ceremonies worsened their precarious situations, forcing them to sell assets (especially gold) or go into further debt. Households who had recently held the marriage of their daughter, especially those that required assistance to contribute gold ornaments to dowries, were put in particularly difficult situations as they owed large debts to their kin. Yet, for many, stopping the chain of gifts and counter‐gifts, thus rupturing social relationships even temporarily, was inconceivable.

The obligation to gift also became an important reason for taking microfinance loans post‐moratorium. For instance, two Dalit women — who, for months, reported being overwhelmed by loans they were unable to repay and insisting they would not take any new ones — both applied for new loans in January 2021. Both had borrowed expensively — at 4 per cent per month and 0.5 per cent per day — and had already made gifts they owed. They were now struggling to secure cheaper credit to minimize interest payments to their current lenders. MFIs became their best option.

The crisis further exposed the extent to which both the position in the life cycle and the quality of the social network constituted crucial factors determining participants’ ability to navigate financial challenges (see also Guérin et al., 2019). In the context of a breakdown of access to formal finance, the social ties ingrained in ceremonial credit represented an undergirding infrastructure to remain part of, at all costs.

## CONCLUSION

This article has explored the unequalizing impacts of the COVID‐19 lockdown on microfinance borrowers in Tamil Nadu, India. We argue that understanding financial inclusion as political, social, relational and material *infrastructures* which are characterized and reproduced by everyday social relations allows us to demonstrate how the crisis has further exposed the limits of for‐profit financial inclusion interventions. Despite massive efforts to build a financial inclusion infrastructure, the relational infrastructure — the myriad of informal exchanges, from daily debt to long‐term ceremonial exchanges — remains the primary (albeit unequal) form of security and protection for informal workers. By prioritizing lending restrictions and loan repayments, the response of the microfinance industry to the crisis in the three study villages, and in India more broadly, revealed the exclusionary dimensions of such infrastructures. The drive to both reduce the impacts of the crisis for microfinance institutions and maintain their capacity to extract revenues from/in the future came at the expense of debtors’ livelihoods and survival strategies. What's more, relief schemes and programmes underpinning the financial inclusion infrastructures did not live up to their promises, with the distribution of emergency loans and digital social benefits proving to be highly selective, oriented by political concerns, demarcated along caste and class lines as well as, in some cases, sources of capture.

Our evidence also suggests that the unequal breakdown of financial inclusion infrastructures during COVID‐19 has reinforced the significance of already‐existing and hierarchical circuits of exchange and forms of solidarity. The pawning of gold as well as reciprocal payments through life‐cycle events became particularly significant during and after the lockdown period. They provided rare opportunities to access liquidity and repay microfinance loans, thus generating both social and material value. However, we have shed light on how the fragility of a credit‐based economy is further exposed in times of crisis. Whether it is microcredit, informal debt or gifts/counter gifts, poor households are drowning in debt. Whilst in ‘normal’ times, some of these debts may represent a way to project oneself into the future, the crisis has revealed the devastating consequences of such precarious forms of inclusion, for social as well as financial security. In addition to being intertwined, formal and informal financial infrastructures are riven with inequality. Far from creating a safety net of solidarity, social and relational infrastructures of finance are also imbued with power asymmetries, with caste, class and gender all (re)shaping ties between debtors and creditors. The breakdown of financial inclusion infrastructures reverberates through the underlying social structure, reinforcing social hierarchies and dynamics of destitution.

At the time of writing, the possibility of a massive payment default cannot be excluded. Whether the Indian microfinance industry has withstood the crisis, and the extent to which the cost of it will be borne by borrowers, remains to be seen. Above all, the COVID‐19 crisis and the subsequent lockdown have highlighted the extent to which not more financial inclusion but rather more social protection is urgently required for the working poor in India.

## Footnotes

1 Dalit is the term used to represent historically oppressed castes.

2 Interview Veena Mary, Selvanagar (by phone from Pondicherry), 4 September 2020. Note: all names of individuals and villages have been changed to protect the identity of the respondents.

3 At first microfinance providers had been permitted to charge compound interest on the outstanding balance of loans on which the moratorium had been availed, for the period when payments were on hold. This meant having to pay over 3 per cent interest for every month borrowers chose to delay repayment, amounting to a total additional interest of 15 per cent for five months. This was to be added to future payments, in addition to the standard rate of 25 per cent per annum being charged already. Finally, after a court battle, the compound interest was revised to simple interest at the end of October 2020 and the difference was refunded. Borrowers were still charged interest for the moratorium but not interest on the accruing interest in addition to this. In the end, they paid 10 per cent interest on the total outstanding amount for this period.

4 Here we refer to the notion that microfinance loans are repaid through the commitment of future income streams to debt repayments. As such, microfinance institutions are able both to capture future income, and to do so in a manner that is temporally rigid, with debt repayments being quite immovable.

5 Interest accrual is the accumulation of interest based on principal outstanding (i.e. the remaining amount of the original loan), when repayments are not made.

6 Interest capitalization, or interest on interest, refers to the charging of interest on top of the amount of accrued interest.

7 The village‐level governing body.

8 For instance, Interview Chinathaayi, Pudur, 9 March 2020.

9 In our interviews, data on microcredit repayment were available for 31 households: almost all non‐Dalits have repaid at least part of their microcredit (10 out of 11) while most Dalits have not (17 out of 21).

10 Interview Parvati, Pudur (by phone from Bangalore), 7 November 2020.

11 Interview Veronica, Selvanagar, 5 March 2021.

12 Interview Vanitha, Pudur, 13 March 2021.

13 Demonetization refers to the government's attempt to formalize the economy by banning the 500 and 1,000 rupee banknotes, the two highest value notes in circulation.

14 Interview Elangovan, Manathur (by phone from Pondicherry), 13 May 2020.

15 Interview Sumitra, Manathur (by phone from Bangalore), 10 June 2020.
